# Sleep and schizophrenia: From epiphenomenon to treatable causal target

**DOI:** 10.1016/j.schres.2019.11.014

**Published:** 2020-07

**Authors:** Felicity Waite, Bryony Sheaves, Louise Isham, Sarah Reeve, Daniel Freeman

**Affiliations:** a Department of Psychiatry, University of Oxford, UK; b Oxford Health NHS Foundation Trust, UK; c Sleep and Circadian Neuroscience Institute, University of Oxford, UK; d University College London, UK

**Keywords:** Insomnia, Schizophrenia, Psychosis, Hallucinations, Delusions, Treatment

## Abstract

**Background:**

Sleep disturbance is a common clinical issue for patients with psychosis. It has been identified as a putative causal factor in the onset and persistence of psychotic experiences (paranoia and hallucinations). Hence sleep disruption may be a potential treatment target to prevent the onset of psychosis and reduce persistent psychotic experiences. The aim of this review is to describe developments in understanding the nature, causal role, and treatment of sleep disruption in psychosis.

**Method:**

A systematic literature search was conducted to identify studies, published in the last five years, investigating subjective sleep disruption and psychotic experiences.

**Results:**

Fifty-eight papers were identified: 37 clinical and 21 non-clinical studies. The studies were correlational (*n* = 38; 20 clinical, 18 non-clinical), treatment (*n* = 7; 1 non-clinical), qualitative accounts (*n* = 6 clinical), prevalence estimates (*n* = 5 clinical), and experimental tests (*n* = 2 non-clinical). Insomnia (50%) and nightmare disorder (48%) are the most prevalent sleep problems found in patients. Sleep disruption predicts the onset and persistence of psychotic experiences such as paranoia and hallucinations, with negative affect identified as a partial mediator of this relationship. Patients recognise the detrimental effects of disrupted sleep and are keen for treatment. All psychological intervention studies reported large effect size improvements in sleep and there may be modest resultant improvements in psychotic experiences.

**Conclusions:**

Sleep disruption is a treatable clinical problem in patients with psychosis. It is important to treat in its own right but may also lessen psychotic experiences. Research is required on how this knowledge can be implemented in clinical services.

## 1 Introduction

“*When I'm tired, everything is worse*”

(Patient with psychosis, Waite et al., 2016a, p.186)

Since the first descriptions of schizophrenia sleep dysfunction has been a feature of patient, family, and clinician accounts (see Kraeplin, 1919; Birchwood et al., 1989; Yung and McGorry, 1996). Despite this, sleep problems have been overlooked as a treatment target. Historically, sleep disruption has been seen as a non-specific epiphenomenon or secondary consequence of a core psychotic disturbance. Over the past ten years there has been increasing interest in a different view: that sleep dysfunction may be a potential causal mechanism in the onset and persistence of psychotic experiences (Anderson and Bradley, 2013; Freeman et al., 2009; Reeve et al., 2015; Soehner et al., 2013; Waters et al., 2017). This paradigm shift identifies sleep disruption as a potential novel treatment target for psychotic symptoms. It also provides a route to determine causality: if successful treatment of sleep problems results in improvements in psychotic experiences then a causal relationship is demonstrated. Our previous review found that sleep disturbance and psychotic experiences co-occur (Reeve et al., 2015), but the direction of effect and underlying mechanisms were yet to be established. Given the recent work on sleep and psychosis, particularly treatment innovation, it is timely to review the current status of the evidence and consider the implications for clinical practice.

In patients with psychosis sleep problems are very common. For example, in a survey of 1809 patients with non-affective psychosis attending NHS mental health services, 50.1% had clinically significant levels of insomnia (Freeman et al., 2019). Disturbances in sleep architecture and circadian systems are also typically found in patients (Cosgrave et al., 2018). Indeed the clinical picture is complex, with comorbidity of sleep disorders normal rather than the exception (Reeve et al., 2019b). Patient accounts highlight an interaction between sleep difficulties and psychotic experiences and describe the negative impact on functioning (Faulkner and Bee, 2017; Waite et al., 2016a; Waite et al., 2018). Clinicians certainly recognise the prevalence and importance of sleep problems, but in current practice formal assessment infrequently occurs and the provision of recommended treatment is extremely limited (Rehman et al., 2017; Reeve et al., 2019b). Yet there is a clear desire for treatment: three-quarters of patients with psychosis who have insomnia would like treatment to improve their sleep (Freeman et al., 2019).

In international guidelines the recommended first line treatment for insomnia is cognitive behavioural therapy (CBTi) (for example, National Institute for Health and Care Excellence, 2015; Qaseem et al., 2016; Wilson et al., 2019). CBTi targets the subjective sleep disturbance which characterises insomnia. It has a well-established evidence base demonstrating effectiveness in treating insomnia (without comorbid psychosis), for example a recent meta-analysis of 87 randomised controlled trials (RCT) found large effect size improvements in self-reported insomnia symptoms (hedges g = 0.98) (van Straten et al., 2018). In recent years, researchers have begun to investigate the applicability, feasibility, and effectiveness of CBTi in people with psychotic experiences in the general population (Freeman et al., 2017), individuals at ultra-high-risk of psychosis (Bradley et al., 2018, patients with persistent psychotic experiences (e.g. Freeman et al., 2015; Hwang et al., 2019; Chiu et al., 2018;) and patients admitted to acute psychiatric inpatient care (Sheaves et al., 2018a). This review aims to provide an overview of sleep disruption and its treatment across this spectrum of psychosis severity. Given previous reviews on the occurrence of sleep disruption and its contribution to psychotic experiences (see Cosgrave et al., 2018; Davies et al., 2017; Reeve et al., 2015) the main focus of this review will be on treatment developments in the past five years. Consistent with treatment guidelines this review will focus on evidence-based psychological interventions. By conducting a literature review of the studies published in the last five years investigating sleep disruption and psychotic experiences, we sought to address the following questions:

1. What is the experience of sleep disruption and its treatment in patients experiencing psychotic symptoms?
2. Do subjective sleep disturbance and psychotic experiences co-occur across this spectrum of psychosis severity?
3. Are psychological interventions for sleep disturbance (CBT) applicable, feasible, and effective on a) subjective sleep problems, b) psychotic experiences?

## 2 Method

A search was carried out on PubMed for English language papers published in peer-reviewed journals containing the following terms: ((sleep OR insomnia OR dream* OR nightmare*) AND (Delus* OR Hallucinat* OR Psychosis OR *Schizophren* OR Schizotyp*)), published in the last five years (search conducted on 5th August 2019). Literature on non-human studies was not included. The search criteria were consistent with our earlier review (Reeve et al., 2015) to enable a focus on recent findings.

Both qualitative and quantitative methodologies were included. Papers relating primarily to dementias or other neurological conditions, bipolar disorder, or affective psychoses (including post-partum psychosis) were excluded. Previous reviews were excluded. For quantitative studies, a specific measure of subjective sleep disturbance was required. There are elevated rates of obstructive sleep apnea in patients with psychosis (24%) (see Annamalai et al., 2015) and an established respiratory treatment, however this is outside the scope of this review.

**Fig. 1:**
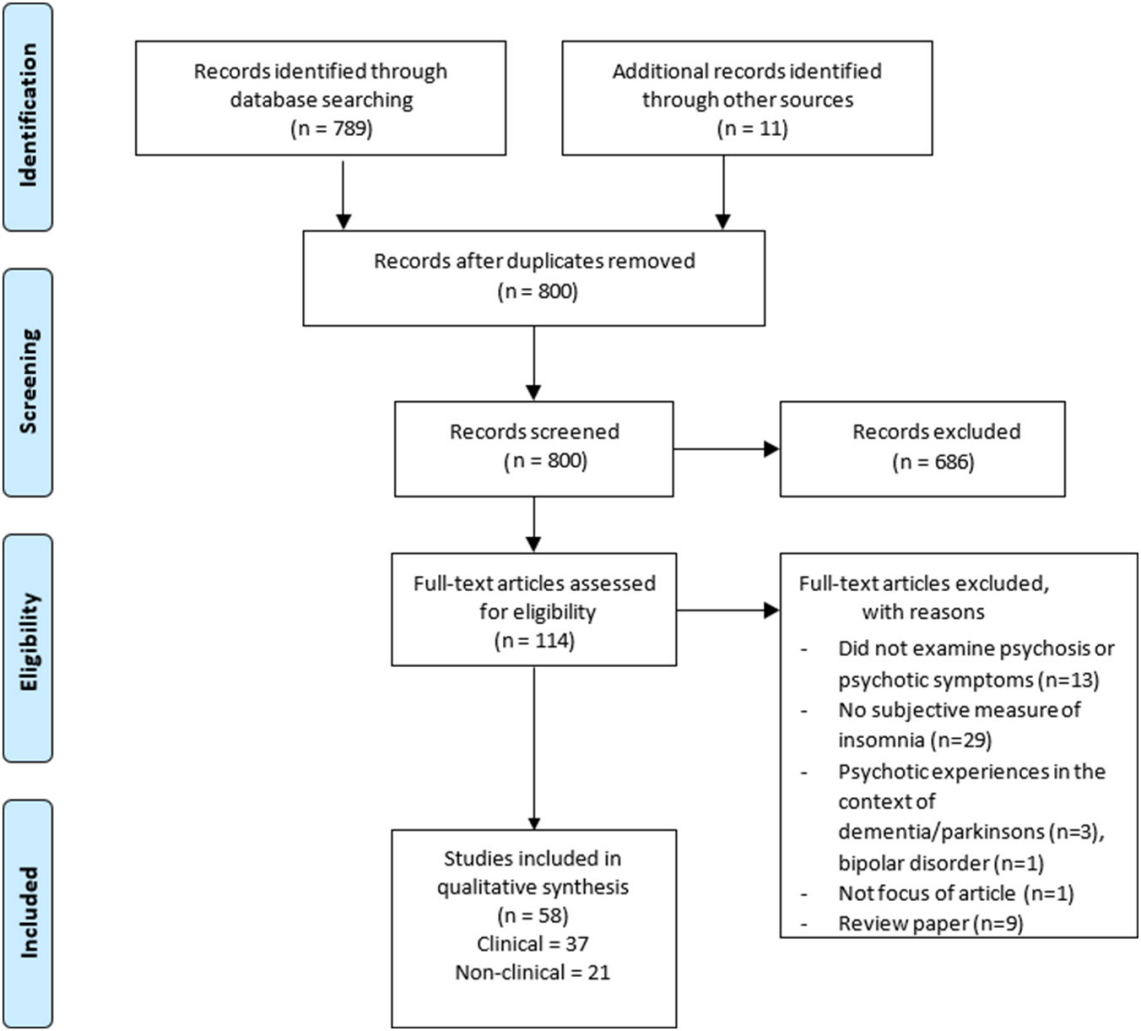
PRISMA flow diagram of the systematic review process.

This search revealed 789 papers. Titles and abstracts were scanned, and if appropriate the whole paper, in order to ascertain the inclusion and exclusion criteria. The reference lists of relevant papers were also scanned for further citations, which provided an additional 11 papers. See Fig. 1 for a PRISMA flow diagram of the systematic review process.

## 3 Results

Fifty-eight papers were included in the review. Thirty-seven papers concerned clinical samples (8 ultra-high-risk, 3 first-episode psychosis, 22 schizophrenia, 4 acute inpatients) and 21 concerned non-clinical samples. Within the clinical samples there were six qualitative studies, five studies assessed the prevalence of sleep disturbance, 20 studies provided correlational data, and six studies evaluated psychological treatment. In the non-clinical studies, 18 studies provided correlational data, two were manipulation tests, and one study concerned treatment.

### 3.1 The patient experience of sleep disruption and its treatment

“*…because, the voices, and there's too many things on your mind and you get anxious and stuff like that but it's...yeah, it is quite hard to get to sleep, it's stressful.*”

(patient 15; Faulkner and Bee, 2017)

“*When I'm tired it gets worse because I don't have the strength to fight the voices as much*”.

(patient 1; Waite et al., 2016a)

Across the spectrum of severity of psychosis there is a striking consistency in patient accounts of the nature of sleep disturbance, the interaction with psychotic experiences, the negative impact on functioning, and treatment preference (Chiu et al., 2016; Faulkner and Bee, 2017, Faulkner and Bee, 2016; Waite et al., 2016a; Waite et al., 2018; Waters et al., 2015).

Patients describe how sleep can be disrupted by psychotic experiences, for example: “if I'm having voices and stuff I find it harder to sleep, my mind's racing” (3, Chiu et al., 2016); “These voices like to see me awake and agitated.” (13, Faulkner and Bee, 2017). Patients also highlight the reverse: that sleep problems contribute to the occurrence of psychotic experiences. Indeed, patients describe the reciprocal nature of sleep problems and psychotic experiences as a “nightmare you can't get out of” (4, Waite et al., 2016a). Yet this also indicates a self-identified avenue for improvement: "sometimes my voices would be better as well from sleeping" (2, Waite et al., 2016a). In young people at ultra-high-risk of psychosis, sleep disturbance was typically characterised by disrupted sleep timing (Waite et al., 2018). In contrast, patients with persistent psychotic experiences often described being reliant on hypnotic medication or using neuroleptic medication as a route to initiate sleep: “knocking yourself out” (Faulkner and Bee, 2017, p.8).

Despite wanting better sleep, the accounts highlight a sense of hopelessness and limited expectation concerning treatment outcome. There were often beliefs that sleep simply cannot be changed: “I just seem to be at the mercy of it you know. I just don't really have any control over it really. It's just sometimes it happens for me, and sometimes it doesn't.” (5, Chiu et al., 2016). This sense of resignation was more profound in patients with persistent psychosis, for example: “I know I'm going to be like this forever, there's no point in telling a lie to myself, I know I'll always have problems sleeping" (2, Faulkner and Bee, 2017). Yet it was already present in many young people at ultra-high-risk of psychosis when offered a sleep intervention: “I didn't think it would work at first” (8, Waite et al., 2018).

Those patients who had received a psychological sleep intervention described important clinical change and valuing the therapy: “It was really, really good. Really good. I was really impressed. I didn't think it was going to help or anything. I am really impressed” (10, Waite et al., 2016a). For some, the process of implementing new strategies was challenging: “It's hard to try and change, to try and get myself motivated” (7, Waite et al., 2018). Yet, for others, treating sleep problems was identified as a route to improving wider mental health. With some patients explicitly describing an improvement in psychotic symptoms with improved sleep: “since I've been sleeping better, my, visual things have like stopped” (8, Waite et al., 2018).

### 3.2 The prevalence of sleep disturbance in patients

The patient accounts of sleep disturbance are consistent with the empirical data. Rates of sleep disturbance are elevated across the spectrum of severity of psychotic experiences. Studies indicate that 75–80% of patients at ultra-high-risk (Poe et al., 2017), those who have experienced a first episode (Ma et al., 2018; Reeve et al., 2019b), and patients with a diagnosis of schizophrenia (Laskemoen et al., 2019) are experiencing subjective sleep disturbance. There were no studies in the past five years reporting prevalence in acute settings. When specific sleep disorders are assessed using self-report or clinician interview measures the rate of insomnia is approximately 50% (Freeman et al., 2019; Laskemoen et al., 2019), the rate of weekly nightmares 55% (Sheaves et al., 2015), and the rate of hypersomnia 30% (Laskemoen et al., 2019) in patients with non-affective psychosis. Only one study has conducted formal diagnostic sleep interviews in patients with psychosis (Reeve et al., 2019b). In a sample of 60 patients with early psychosis, insomnia (50%) and nightmares (48%) were the most common disorders. Sleep disorders were not only highly prevalent but frequently comorbid: the average number of sleep disorders per patient was 3.3 (Reeve et al., 2019b).

### 3.3 Sleep disturbance as a causal factor for psychotic experiences

#### 3.3.1 Correlational data

Our previous review found a robust association between sleep dysfunction and psychotic experiences (Reeve et al., 2015), especially between insomnia and paranoia. The current review found 38 recent studies: 18 general population (Table 1) and 20 clinical samples (Table 2) reporting an association between sleep disruption and psychotic experiences.

**Table 1:** Non-clinical studies.

Reference	Design	Sample	n	Participant characteristics	Measure of sleep disturbance	Measure of psychotic experiences	Key finding
Meyhöfer et al., 2017	Manipulation (sleep deprivation) Within subjects design	General population	32	16 male (50%), mean age 23.97 years (SD = 3.46)	Sleep diary	PSI	Sleep deprivation induced psychosis-like experiences such as hallucinations, cognitive disorganisation and negative symptoms.
Reeve et al., 2018a	Manipulation (sleep restriction) Within subjects design	General population	68	46% male, mean age 22.5 years (SD = 3.4)	Sleep diary and actigraphy	SPEQ	Compared to the control condition, participants in the sleep loss condition reported significant increases in paranoia, hallucinations and cognitive disorganisation. Changes in psychotic experiences were mediated by changes in negative affect.
Andorko et al., 2018	Cross sectional	Students	409	49.4% male, mean age 20.10 years (SD = 3.22)	ISDI	PQ-B	Nightmares and fragmented sleep were both significantly associated with the presence of psychotic-like experiences.
Cosgrave et al., 2018	Cross sectional	Students	43	44.2% male, mean age 23.9 years, (SD = 3.6) in the control group and 22.8 years, (SD = 3.2) in the experimental group.	PSQI, ISI, actigraphy	PQ-16	Objective and subjective sleep measures interact to predict the highest risk of psychotic experiences. The combination of perceived poor sleep and actual lack of sleep predicts the greatest risk for psychotic experiences.
Ered et al., 2018	Cross sectional	Students	2687	26% male, mean age 20.22 years (SD = 3.21)	PSQI	PQ	The relationship between psychotic-like experiences and sleep quality was mediated by symptoms of depression and PTSD, suggesting that treating symptoms of depression and PTSD could improve multiple domains of psychotic illness.
Hennig and Lincoln, 2018	Cross sectional	Healthy volunteers (adolescents)	61	50.8% male, mean age 15.1 years (SD = 1.1)	Sleep diary and actigraphy	Six items from the SPEQ	Shorter sleep duration and more dreaming predicted paranoid symptoms, but paranoid symptoms did not significantly predict sleep parameters. Positive and negative affect partially mediate the effect of sleep duration on paranoid symptoms.
Peña-Falcón et al., 2019	Cross sectional	General population	177	35.6% male, mean age 31.17 years (SD = 12.85)	PSQI, Iowa sleep experiences survey	Launay–Slade Hallucination Scale-revised, DES.	There was a significant positive association between quality of sleep and hallucination proneness, dissociation and unusual sleep experiences. Sleep quality and hallucination proneness was fully mediated by dissociation and unusual sleep experiences.
Rehman et al., 2018	Cross sectional	General population (healthy volunteers)	Study 1, 401; study 2, 402	Study 1: 23.2% male, mean age 24 (SD = 8.1); Study 2: 28.4% female, mean age 24 years (SD = 10.8)	PSQI	GPTS-B; CAPS.	Study 1: The relationship between sleep quality and paranoia was partially mediate by alexithymia, perceptual anomalies and negative affect. Study 2: The relationship between sleep quality and paranoia was fully mediated by negative affect, perceptual anomalies and alexithymia.
Andorko et al., 2017	Cross sectional	Students	409	49.1% male, mean age 20.09 years (SD = 3.22)	ISDI	PQ-B	Fragmented sleep, night anxiety and disturbed sleep all significantly correlated with psychotic-like experiences.
Moritz et al., 2017	Longitudinal	General population	2357	40.8% male, mean age 46.9 years (SD = 14.5)	PHQ-9	18-item PCL	Paranoia predicted sleep dysfunction six months later, but not the reverse.
Rek et al., 2017	Cross sectional	General population	846	11% male, mean age 44 years (SD = 15.9)	NSS, ISIS, MCTQ	SPEQ, CDS	When controlling for negative affect, nightmare occurrence was associated with high levels of worry, depersonalisation, hallucinatory experiences, paranoia, and sleep duration. Nightmare severity was associated with higher levels of worry, depersonalisation, hallucinatory experiences, and paranoia.
Scott et al., 2017	Cross sectional	General population	389	24% male, mean age 36.49 (SD = 12.76)	8-item insomnia subscale of the Sleep-50 questionnaire	GPTS-B, DASS-21	The findings point to an association between perceived (but not objective) difficulties initially falling asleep (but not maintaining sleep) and paranoid thinking; a relationship that is fully mediated by negative affect.
Oh et al., 2016	Cross sectional	General population (national epidemiological survey)	2304	Gender and age not reported.	DSM-IV criteria for sleep disturbance	WHO-CIDI 3.0 Psychosis Screen	People with sleep disturbances lasting 2 weeks or more over the past 12 months were significantly more likely to report at least one psychotic experience over the same time frame, when compared to people without any sleep problems.
Sheaves et al., 2016a	Cross sectional and longitudinal	General population (two national epidemiological surveys)	2000 dataset: 8580 2007 dataset: 7403 Longitudinal dataset: 2406	2000 dataset: 46.8% male, mean age 43.87 (SD = 0.23) 2007 dataset: 48.6% male, mean age 46.35 (SD = 0.03) Longitudinal dataset: 49.3% male, mean age: 43.44 (SD = 0.49)	Clinical Interview (CIS-R)	PSQ	Insomnia was associated with hallucinations in both cross-sectional datasets. Mild sleep problems were associated with 2–3 times greater odds of reporting hallucinations, whilst chronic insomnia was associated with four times greater odds. These associations remained significant, although with smaller odds ratios, after controlling for depression, anxiety and paranoia.
Sheaves et al., 2016b	Cross sectional	Undergraduate and postgraduate students	1403	44.4% male Median age: 21 (interquartile range = 20–23)	SCI, retrospective Dream Log (adapted from Levin and Fireman), MCTQ, SJL.	SPEQ, DASS-21.	Insomnia, nightmares, and circadian phase delay are associated with increased subsyndromal psychiatric symptoms in young people. Each is a treatable sleep disorder and might be a target for early intervention to modify the subsequent progression of psychiatric disorder.
Chung et al., 2015	Longitudinal cohort	General population (Taiwan's National Health Insurance Research Database)	30,670	41.1% male, age reported by subgroups	Diagnosis of insomnia, prescription for sedative-hypnotics at a defined daily dose of at least 30 per year.	Diagnosis of psychiatric disorder.	People with insomnia taking sedative-hypnotic prescriptions had an elevated risk of developing psychiatric disorders compared to those without insomnia or a sedative-hypnotic prescription.
Jeppesen et al., 2015	Cross sectional	11–12 year old population cohort sample (Copenhagan child cohort 2000)	1632	Gender and age not reported.	Self-reported sleep problems and patterns in a structured interview.	K-SADS-PL-semi structured interview.	Sleep problems increased the risk of psychotic experiences, after controlling for gender, puberty and other mental disorders. Psychotic experiences are particularly prevalent in the context of sleep disturbance and affect dysregulation.
Koyanagi and Stickley, 2015	Cross sectional	General population (WHO epidemiological survey including 70 countries)	26,1547	Gender and age reported by country.	Single question problem sleeping (falling asleep, waking up frequently, or too early), 5-point scale (none -extreme).	CIDI 3.0 positive psychotic symptoms.	Sleep problems associated, in a dose-response fashion, with psychotic symptoms in almost all countries.
Taylor et al., 2015	Cross sectional	16 year old twin pairs from population cohort (TEDS)	5076 pairs of twins	53% male, age 16 years	PSQI, ISI	SPEQ	Shared genetic and environmental mechanisms for psychotic experiences and sleep disturbance. Association remained after controlling for negative affect.
Thompson et al., 2015	Longitudinal.	18 year old population cohort sample (ALSPAC)	4720	43.5% male (*n* = 2666) and age 18 years	Postal questionnaire (completed by mother) at age2.5, 3.5, 4.75, 6.75, 9 years. Experience of nightmares, night terrors and sleepwalking was assessed using a semi-structured interview at age 12 years.	PLIKSi at age 12 and 18 years.	Nightmares at 12 were a significant predictor of psychotic experiences at 18, remained after adjustment for possible confounders and psychotic experiences at 12 years.

Table 1 key: CAPS = Cardiff anomalous perceptions scale; CDS = Cambridge depersonalisation scale; CIS-R = Clinical interview schedule - revised; DASS-21 = Depression, anxiety, and stress scale; DES = Dissociative experience scale; GPTS = Green paranoid thoughts scale; ISDI = Iowa sleep disturbances inventory; ISI = Insomnia severity index; MCTQ = Munich chronotype questionnaire; NSS = Nightmare severity scale; PCL = Paranoia checklist; PHQ-9 = Patient health questionnaire; PLIKSi = Psychosis-like symptoms semi-structured interview; PSI = Psychotomimetic states inventory; PQ = Prodromal questionnaire (PQ-B brief; PQ-16 version); PSQ = Psychosis screening questionnaire; PSQI = Pittsburgh sleep quality index; SCI = Sleep condition indicator; SJl = Social Jet Lag; SPEQ = Specific psychotic experiences questionnaire.

**Table 2:** Correlational studies with clinical samples.

Reference	Design	Sample	n	Participant characteristics	Measure of sleep disturbance	Measure of psychotic experiences	Key finding
Goines et al., 2019	Longitudinal	Youth with clinical high risk (CHR)	1020	CHR (n=740), Gender (male = 424), Age (m = 18.5, SD = 4.26). HC (n=280), Gender (male = 141), Age (m = 19.7, SD = 4.67).	SOPS (G1 Sleep Item).	SIPS, SOPS	Positive association between sleep problems and attenuated psychotic symptom severity. Sleep problems closely associated with suspiciousness. Depression mediated the cross-sectional association between sleep problems and paranoid symptoms only.
Reeve et al., 2019b	Longitudinal	Individuals at risk of psychosis	160	Gender (male = 98), Age (m = 20.9, SD = 4.2).	Self-reported sleep duration from EPQ interview.	CAARMS	Association found between shorter sleep duration and increased positive symptoms, but not cognitive disorganisation. Longitudinal relationships did not remain significant when controlling for previous severity of psychotic symptoms.
Bird et al., 2017	Longitudinal, Observational	Help-seeking adolescents reporting paranoid thoughts.	34	Gender (male = 6), Age (m = 14.9, SD = 1.25).	ISI	GPTS, PANSS (suspiciousness/persecution item), SPEQ.	Insomnia found to be a significant predictor of paranoia persistence.
Lunsford-Avery et al., 2017a	Cross-sectional	Adolescents at ultra-high risk (UHR)	62	Gender (male = 37), Age (m = 18.93, SD = 1.67).	PSQI	SIPS, SCID	Self-reported sleep problems were associated with impaired procedural learning rate.
Lunsford-Avery et al., 2017b	Longitudinal	Adolescents with clinical high risk (CHR)	66	CHR (n=34), Gender (male = 15), Age (m = 18.79, SD = 1.93) HC Gender (male = 16), Age (m = 17.75, SD = 2.79)	Actigraphy, Sleep/Activity Diary	SIPS, SCID	In CHR participants, circadian disturbances were associated with psychotic symptom severity and predicted symptom severity and psychosocial impairment at 1-year follow-up.
Poe et al., 2017	Longitudinal	Help-seeking clinical high risk (CHR) patients.	260	CHR (n=194), Gender (male = 142), Age (m = 20.0, SD = 3.8). HC (n=66), (male = 42), Age (m = 21.9, SD = 3.6)	SIPS (G1 Sleep Items)	SIPS	Rates of sleep disturbance were significantly elevated in patients. Sleep disturbance was associated with increased positive and negative symptoms and worse overall functioning.
Lunsford-Avery et al., 2015	Longitudinal	Adolescents with ultra-high risk (UHR)	67	UHR (n=31), Gender (male = 19), Age (m = 18.73, SD = 1.89). HC (n=36), Gender (male = 16), Age (m = 17.85, SD = 2.62).	Actigraphy (TST, WASO, sleep efficiency and total movement counts), Sleep/activity diary, PSQI	SIPS, SOPS, SCID	Patients displayed reduced efficiency, disrupted continuity, and increased movements during sleep compared to HC. This behaviour was associated with increased positive symptoms at baseline.
Kasanova et al., 2019	Longitudinal (Experience sampling) Mediation analysis	Acutely paranoid patients with a psychotic disorder (AP), non-paranoid patients with a psychotic disorder (NP) and individuals with high schizotypy traits (ST).	115	AP (n = 42) NP (n = 32) ST (n = 41)	Momentary sleep quality: "I slept well last night" (7-point likert scale)	PANSS CAPE-42 Momentary paranoia (7-point likert scale)	Poor subjective sleep quality predicted elevated paranoia the following morning. This relationship was fully mediated by morning negative affect. No significant association between evening paranoia and poor sleep the following night emerged.
Chung et al., 2018	Cross-sectional	Outpatients with schizophrenia with delayed sleep-wake phase disorder and normal sleep-wake phase	66	Gender (male = 30), Age (m = 44.08, SD = 12.64).	Clinical interview using ICSD-3 criteria for delayed sleep-wake phase disorder. Actigraphy, The consensus sleep diary, CSM, PSQI, ESS	PANSS	Sleep irregularity was associated with positive psychotic symptoms and depressive symptoms.
Reeve et al., 2018b	Mediation analysis, longitudinal, observational	Outpatients with early non-affective psychosis diagnoses.	29	Gender (male = 13), Age (m = 23.55, SD = 3.8).	Sleep-50	SPEQ	Insomnia is a significant predictor of paranoia and hallucinations both within and across time, with the relationships mediated by negative affect.
Subramaniam et al., 2018	Cross-sectional	First-episode psychosis.	279	Gender (male = 142), Age (m = 25.8, SD = 6.2).	ISI,	SCID, PANSS	Insomnia was associated with significant decreases in all QOL domains assessed in the study even after adjusting for confounders.
Hou et al., 2017	Cross-sectional	Patients with schizophrenia	623	Gender (male = 341), Age (m = 47.7, SD = 10.3).	Interview - DIS; DMS; EMA, TST	BPRS	Long sleep was associated with unemployment and use of second-generation antipsychotics.
Kilicaslan et al., 2017	Cross-sectional	Outpatients with schizophrenia	199	Gender (male = 136), Age (m = 40.42, SD = 11.20).	PSQI	SCID, PANSS	Positive association found between subjective sleep quality and positive symptoms.
Li et al., 2017	Cross-sectional	Outpatients with schizophrenia	612	Gender (male = 337), Age (m = 47.7, SD = 10.3).	Interview - DIS; DMS; EMA	BPRS	Insomnia associated with significantly lower quality of life.
Li et al., 2016	Longitudinal, observational	Outpatients with schizophrenia-spectrum diagnoses.	388	Gender (male = 175), Age (m = 41.0, SD = 11.4).	Parasomnia questionnaire	ICD-10 Diagnosis	Insomnia associated with increased incidence of suicidal behaviour.
Noort et al., 2016	Cross-sectional	Outpatients with Schizophrenia (SP, n = 30), outpatients with depression (DP, n = 30) and healthy controls (HC, n = 30).	90	Gender (male = 23), Age (m = 41.20, SD = 10.78).	PSQI	ICD-10 Diagnosis	There was a significant negative relationship between PSQI score and reading span task scores. No differences found between SP and HC on working memory tasks.
Afonso et al., 2014	Cross-sectional	Outpatients with schizophrenia (SP)	68	SP (n=34) Gender (male = 22), Age (m = 33.8, SD = 8.6). HC (n=34) Gender (male = 19), Age (m = 34.7, SD = 8.3).	Actigraphy, PSQI.	PANSS	Greater sleep disturbance, poor sleep quality and poor quality of life found in patients compared to controls.
Si et al., 2019	Cross-sectional (*Longitudinal analyses do not include sleep item)	Acute inpatients with schizophrenia	602	Gender (male = 289), age (m= 32.4, SD=11.31).	Sleep quality (specific measure or item not detailed).	PANSS	Sleep quality was associated with social functioning.
Langsrud et al., 2016		Acute inpatients (n=49 with schizophrenia, n=28 mood disorders, n=27 substance use, n=20 anxiety, personality or developmental disorders, n=11 organic disorder).	135	Gender (male = 74), age (m= 39.0, SD=15.6).	Sleep diary completed by nursing staff.	Diagnostic category used only. Duration of inpatient admission.	Sleep duration on the night of admission negatively correlated with length of admission.
Chiu et al., 2015	Cross-sectional	55 psychiatric inpatients with psychosis and 66 healthy controls. 25 in each group had insomnia.	121	Inpatients Gender (male = 74.5%), age (19-70) Controls (n=66, Gender (male = 42.4%), age (18-84)	ISI, Thought Control Questionnaire for Insomnia-Revised, Dysfunctional Beliefs and Attitudes about Sleep scale, Sleep Hygiene Knowledge scale, and Beliefs about Causes of Sleep Problems questionnaires	Diagnostic category used only.	Inpatients frequently reported the causes of insomnia to be related to their illness (rather than to their lifestyle factors) and had an incomplete understanding of good sleep habits.

Table 2 key: BPRS=Brief Psychiatric Rating Scale; CAARMS = Comprehensive Assessment of At-Risk Mental States; CAPE-42 = Community Assessment of Psychotic Experience; CSM = The Composite Scale of Morningness; DIS = difficulty initiating sleep; DMS = difficulty maintaining sleep; EMA = early morning wakening; EPQ = Economic Patient Questionnaire interview; ESS = Epworth Sleepiness Scale; G-PTS = Green et al. paranoid thoughts scale; ICSD-3 = International Classification of Sleep Disorders, 3rd Edition; ICD-10 = International Classification of Diseases, 10th Revision; ISI = Insomnia Severity Index; PANSS=Positive and Negative Symptom Scale; PSQI=Pittsburgh Sleep Quality Index; SCID-II=Structured Clinical Interview for DSM-IV disorder; SIPS= Structured Interview for Prodromal Symptoms); SOPS= Scale of Prodromal Symptoms; SPEQ = Specific Psychotic Experiences Questionnaire (Ronald et al., 2014); TST = Total Sleep Time; WASO = wake time after onset.

##### 3.3.1.1 General population studies

The findings from these recent studies robustly confirm the cross-sectional association between sleep dysfunction and psychotic experiences. For example*,* an analysis of data from over a quarter of a million people in the general population found that insomnia significantly increased the likelihood of reporting at least one psychotic symptom (OR = 2.41; 95% CI 2.18–2.65) (Koyanagi and Stickley, 2015). In addition to the evidence for insomnia, an online study with 846 participants from the general population found that, even after controlling for PTSD and negative affect, both nightmare occurrence and severity were associated with hallucinatory experiences and paranoia (Rek et al., 2017).

Several studies provide insight into the temporal relationship using longitudinal and experience sampling method (ESM) designs. For example, in a sample of 4720 young people, nightmares at age 12 predicted psychotic-like experiences (PLE) at age 18 (OR = 1.62, 95% CI 1.19–2.20) (Thompson et al., 2015). In an ESM study of 61 adolescents, shorter sleep duration predicted paranoia, but paranoid symptoms did not predict sleep parameters (Hennig and Lincoln, 2018).

Within correlational studies, a number of potential mechanisms have been investigated. For example, an online study with 2678 students found that depression and PTSD symptoms, but not anxiety symptoms, mediated the relationship between poor sleep and PLEs (Ered et al., 2018). This study examined positive symptoms, but did not report the findings for individual psychotic experiences. Structural equation modelling of a sample of 348 students, recruited in an online study, found an association between subjectively delayed sleep onset and persecutory ideation that was fully mediated by negative affect (Scott et al., 2017). However there are obvious limitations of cross-sectional mediation analyses (see Maxwell and Cole, 2007). Mediation tests related to nightmares are lacking. However, negative affect and related processes such as worry are candidate variables given their strong associations with nightmare occurrence and severity (Rek et al., 2017).

##### 3.3.1.2 Clinical studies

In patients at ultra-high-risk of psychosis, sleep disruption has been associated with positive and negative symptoms (Lunsford-Avery et al., 2015; Poe et al., 2017), disrupted cognitive functioning (Lunsford-Avery et al., 2017), severity of psychotic experiences such as hallucinations and delusions (Reeve et al., 2019a), and overall functioning (Poe et al., 2017). In a large sample of 740 ultra-high-risk patients, sleep disruption was positively correlated with attenuated psychotic experiences, especially suspiciousness (Goines et al., 2019). Depression mediated the association between sleep problems and paranoia.

In patients with psychosis, sleep disturbance is associated with poor clinical outcomes (Afonso et al., 2014; Chung et al., 2018; Hou et al., 2017; Kilicaslan et al., 2017; Li et al., 2017). For example, a naturalistic longitudinal study of 388 patients found that insomnia was associated with an increased risk of suicide attempts (Li et al., 2016). In the only study specifically reporting correlational data in a first-episode sample, insomnia was associated with poorer outcomes on all four quality of life domains assessed (Subramaniam et al., 2018).

Only two studies with patients with psychosis reported mediation analyses (Kasanova et al., 2019; Reeve et al., 2018b). In a longitudinal observational study of 29 patients with non-affective psychosis, insomnia predicted the persistence of psychotic experiences over time: a bidirectional relationship was indicated between insomnia and paranoia however insomnia was found to be a stronger predictor of later hallucinations than the reverse direction (Reeve et al., 2018b). Mediation models were tested and found that negative affect (anxiety and depression) mediated the relationship between insomnia and psychotic experiences. This is consistent with the findings of an ESM study, in which a time-lagged mixed multilevel model was applied to distinguish the contribution of poor sleep quality on morning paranoia, and evening paranoid ideation on subsequent sleep quality (Kasanova et al., 2019). Poor sleep quality predicted elevated paranoia, and this was fully mediated by negative affect. However, no significant association was found between evening paranoia and poor sleep quality.

#### 3.3.2 Causal tests

When the amount of sleep is deliberately reduced in experimental manipulation studies, either via total sleep deprivation (Meyhöfer et al., 2017; Petrovsky et al., 2014) or sleep restriction (Reeve et al., 2018a), it results in increases in psychotic experiences. These studies have only been conducted with non-clinical samples, but all three report sleep loss resulting in increased perceptual distortions such as hallucinations. Reeve et al., 2018a also found an increase in paranoia and cognitive disorganisation, but no significant changes in grandiosity. Mediation analyses revealed that changes in psychotic experiences were mediated by changes in negative affect and related processes (including worry and negative self and other cognitions), but not memory impairment. Together these manipulation studies provide strong evidence that sleep disturbance is a contributory cause to paranoia and hallucinatory experiences.

### 3.4 Interventionist-casual tests

“*It sorted out my sleep and I'm a better person for it.*”

(patient 10, Waite et al., 2016a, p.187)

In the last five years, there have been six intervention studies targeting insomnia and one targeting nightmares (Table 3). These studies have typically been designed to not only test whether sleep can be improved but to find out whether psychotic symptoms reduce (an interventionist-causal approach, Kendler and Campbell, 2009). Most studies have focused on patients with persistent psychotic symptoms (*n* = 3 insomnia, *n* = 1 nightmares). There has been one study conducted in an acute inpatient setting and one with young people at ultra-high-risk of psychosis. However, the most robust finding is provided from a large RCT of university students with insomnia (*n* = 3755) (OASIS trial; Freeman et al., 2017).

**Table 3:** Treatment evaluation studies.

Reference	Design	Sample	Intervention	Outcome		
				Treatment uptake	Sleep measures	Mental health measures
Freeman et al., 2017	Single blind randomised controlled trial of digital CBTi+TAU vs TAU alone.	3755 university students (aged 18 or over) with insomnia (≤16 SCI) from 26 universities across the UK. 1043 (28%) male, 2676 (71%) female, 36 (1%) other. Mean age 24.7 years. 1891 treatment, 1864 control. There were no exclusion criteria.	6 sessions. Digital CBTi programme: Sleepio. The interactive programme is available on an online platform. Completion of an initial assessment drives algorithms which personalise the intervention. Treatment techniques include behavioural (eg sleep restriction), cognitive (eg paradoxical intention) and educational (eg sleep hygiene, sleep processes) components.	1302 (69%) completed at least 1 session; 672 (36%) completed 3 sessions; 331 (18%) completed all 6 sessions. Mean sessions Treatment quality/fidelity standardised due to digitised delivery method.	Significant reductions in sleep disturbance at all timepoints. Post treatment (10 weeks) there were large effect size improvements on the SCI (Cohen's d = 1.11). Change in sleep at 3 weeks explained 30% of the intervention effect on paranoia at 10 weeks; change in sleep at 10 weeks accounted for 58% of the treatment effect on paranoia. Change in sleep at 3 weeks explained 21% of the intervention effect on hallucinations post-treatment; change in sleep at 10 weeks accounted for 39% of the intervention effect on hallucinations.	Significant reductions in paranoia and hallucinations at all timepoints. Small effect size changes post treatment for paranoia GPTS-B (d = 0.19) and hallucinations SPEQ-H (d = 0.24). Improvements in depression (PHQ-9), anxiety (GAD-7), prodromal symptoms (Prodromal questionnaire), nightmares (DDNSI), psychological wellbeing (WEMWBS) and functioning (WSAS) were reported. There was a small increase in mania symptoms (Altman-Mania) in the treatment group.
Bradley et al., 2018	Uncontrolled, feasibility case series of adapted CBTi+TAU.	12 young patients (aged 14–24 years) at ultra-high-risk of psychosis (CAARMS attenuated psychosis criteria) with current sleep problems (≥15 ISI or above cut off on the insomnia or CRD subscales of the SLEEP-50). 6 male, 5 female. Mean age 18.9 years (SD = 1.9). No control group.	8 sessions (10 week treatment window). Individual. Treatment techniques targeted both insomnia (eg stimulus control, sleep hygiene, relaxation) and circadian rhythm disruption (eg sleep/wake realignment, daily activity). Treatment adaptations to account for developmental age and sleep architecture during adolescence are outlined.	11 (92%) completed ≥2 sessions. Mean number of sessions 7.36(SD 0.5). Treatment quality and fidelity not reported.	Large effect size improvement in sleep post treatment (12 weeks) and at follow up (16 weeks) on all measures of sleep: ISI (Cohen's d = 6.8); Sleep-50 (d = 1.7); PSQI (d = 2.9).	Small and medium effect size improvement post treatment in paranoia (d = 0.6, GPTS), hallucinations (d = 0.3, SPEQ), depression (d = 0.5, DASS-21), stress (d = 0.8, DASS-21) and anxiety (d = 0.2 DASS-21).
Freeman et al., 2015	Prospective, assessor blind pilot RCT of CBTi+ TAU vs TAU alone.	50 patients with persistent distressing delusions and/or hallucinations (≥2 PSYRATS) in the context of a diagnosis of non-affective psychosis and insomnia (≥15 ISI). 34 (68%) male 16 (32%) female. Mean age 40.9 years. 24 treatment, 26 control.	8 sessions (12 week treatment window). Individual. Treatment techniques included: Psychoeducation, assessment and goal setting, stimulus control, establishing daytime routine and circadian rhythm, sleep hygiene, relaxation, cognitive therapy to address sleep-related beliefs. Treatment manual outlines key techniques and adaptations – see Waite et al. (2016b).	23 (96%) completed >4 sessions. Mean number of sessions 7.3 (SD 1.9). Treatment quality and fidelity assessed.	Large effect size improvement in sleep post treatment (Cohen's d = 1.9 at 12 weeks) and maintained at follow up (d = 1.2 at 24 weeks) as measured on the primary measure the ISI. Consistent findings on other sleep measures including the PSQI.	Small effect size improvements (d = 0.1 to −0.3) in delusions and hallucinations were reported (PSYRATS, GPTS, PANSS). However, the confidence intervals span 0. Therefore, treatment effect estimations range from reducing to increasing delusions and hallucinations. Small to medium effect size improvements in fatigue (MFI), wellbeing (WEMWBS, CHOICE), and quality of life (EQ5D) were reported.
Chiu et al., 2018	Open label trial of CBTi+TAU vs TAU alone.	74 patients with non-affective psychosis attending outpatient clinics, with insomnia symptoms (≥5 PSQI). 39 (53%) male, 35 (47%) female. Mean age 41.4 years. 50 treatment, 24 control. Latent class analysis used to identify three sleep subtypes. Cluster 1 – Classic severe insomnia (44.6%) Cluster 2 – Insomnia with normal sleep duration (37.8%) Cluster 3– Insomnia with hypersomnia (17.6%)	4 sessions (6 week treatment window). Individual. Session outline: Treatment manual outlines key techniques and adaptations – see Waters et al., 2017.	40 (80%) completed ≥2 sessions. Mean number of sessions not reported. Treatment quality and fidelity not reported.	Significant improvements in sleep (PSQI; sleep hygiene behaviours scale; TST; SE; SOL) at 6 weeks (post-treatment). Differences observed by profile: Cluster 1 – Greatest benefits: longer TST and SE. Cluster 2 – blunted treatment response compared to cluster 1; improvements in daytime functioning. Cluster 3 – reduced TST, reduced SOL.	Significant improvements in severity of psychotic symptoms (MINI) and psychological distress (PHQ-4) post -treatment.
Hwang et al., 2019	Non-randomised, assessor blind, evaluation of group CBTi+TAU vs TAU alone.	63 patients of a residential facility with a diagnosis of non-affective psychosis, current (but stable) psychotic symptoms (PSYRATS), and current insomnia (≥15 ISI). 41 (65%) male, 22 (35%) female. Mean age 44.9 years. 31 treatment, 32 control.	4 sessions. Group format (group size 2–9 participants). Treatment components: Psychoeducation, cognitive therapy, sleep hygiene, stimulus control, sleep restriction, sleep diary completion.	Treatment uptake not reported. However, all participants provided outcome and follow-up data. Mean number of sessions not reported. Treatment quality and fidelity not reported.	Significant reductions in sleep dysfunction as measured on the ISI and PSQI at 4 weeks (post-treatment) and 8 weeks (follow up).	No significant change in psychotic symptoms (PSYRATS), nor depression the BDI, or anxiety measured on the anxiety sensitivity index (ASI).
Sheaves et al., 2018a	Assessor blind pilot RCT of sleep treatment (STAC) + TAU vs TAU alone.	40 inpatients on an acute psychiatric ward with self-reported symptoms of insomnia (≥8 ISI). 18 (45%) diagnosis of non-affective psychosis. 40 (100%) male. Mean age 40 years 20 treatment, 20 control.	2-week treatment window. Number of sessions was flexible. Minimum dose defined as 5 sessions. Individual. Sleep treatment at acute crisis (STAC) includes CBTi, sleep monitoring, and light/dark exposure for circadian entrainment. Treatment manual outlines key techniques and adaptations – see Sheaves et al. 2018.	20 (100%) completed treatment. Mean number of sessions 4.8 (SD 0.6). Treatment quality and fidelity not reported.	Large effect size reductions in insomnia (ISI) at post treatment (2 weeks) (d = 0.9) maintained at follow up (12 weeks).	Small improvements in psychological wellbeing (WEMWBS) post treatment (2 weeks) (d = 0.3). Wide confidence intervals for STAC increasing or decreasing psychiatric symptoms (PANSS, BSS, YMRS) Patients in the treatment group were discharged 8.5 days earlier.
Sheaves et al., 2019	Assessor blind parallel group pilot RCT of brief CBT for nightmares (including IRT) + TAU vs TAU alone.	24 patients with weekly nightmares and persecutory delusions in the context of a diagnosis of non-affective psychosis. 14 (58%) male 10 (42%) female. Mean age 41 years. 12 treatment, 12 control.	4 week treatment window. Individual. Core technique was imagery rehearsal training. Additional strategies included: psychoeducation about nightmares, reducing pre-sleep hyperarousal, increasing coping skills, reducing preoccupation with nightmares, stabilising REM sleep.	12 (100%) completed treatment. Mean number of sessions 8.6 (SD 1.5). Treatment quality and fidelity not reported.	Large effect size reductions in nightmares (DDNSI) and insomnia (SCI) at post treatment (4 weeks) (DDNSI d = −1.06; SCI d = −1.4) maintained at follow up (8 weeks).	Post-treatment improvements were observed in paranoia (GPTS), affective symptoms (DASS-21), dissociation (DES-B), and emotional wellbeing (WEMWBS). There were no changes in hallucinations (CAPS) or activity levels (time budget).

Table 3 key: BDI = Beck depression inventory; BSS = Beck suicide scale; CAARMS = Comprehensive assessment of at-risk mental states; CAPS = Cardiff anomalous perceptions scale; CHOICE = Choice of outcome in CBT for psychoses; CRD = Circadian rhythm disruption; DASS-21 = Depression, anxiety and stress scale; DES = Dissociative experiences scale; DDNSI = Disturbing dream and nightmare severity index; GAD-7 = Generalised anxiety disorder assessment; GPTS = Green et al. paranoid thoughts scale; MFI = Multidimensional fatigue inventory; PANSS = Positive and negative syndrome scale; PHQ-9 = Patient health questionnaire; PSQI = Pittsburgh sleep quality index; PSYRATS = Psychotic symptom rating scales; RCT = Randomised controlled trial; SCI = Sleep condition indicator; SE = Sleep Efficiency; SOL = Sleep Onset Latency; SPEQ = Specific psychotic experiences questionnaire; STAC = Sleep treatment at acute crisis; TAU = Treatment as usual; TST = Total sleep time; WEMWBS = Warwick-Edinburgh mental wellbeing scale; WSAS = Work and social adjustment scale; YMRS = Young mania rating scale.

The goal of the OASIS trial was to definitively test whether there is a causal association between insomnia and paranoia and hallucinations (Freeman et al., 2017). Students with self-reported insomnia were randomised to receive either a digital CBTi intervention in addition to usual care or usual care alone. Post-treatment, there were large effect size reductions in insomnia (SCI cohen's d = 1.1) and small effect size improvements in paranoia (GPTS-B d = 0.19) and hallucinations (SPEQ-H d = 0.24) in the CBTi group compared with usual care. Those who received the digital sleep treatment were less likely to meet ultra-high-risk criteria (indicated by a score ≥ 6 on the 16-item prodromal questionnaire) post treatment. Parallel analyses in the opposite direction indicated that changes in psychotic experiences explained a much smaller (approx. 3%) proportion of change in sleep. This study provides the most definitive evidence to date that sleep treatment can improve psychotic experiences and that insomnia is a contributory causal factor in paranoia and hallucinations. A key limitation is that the study was not in a clinical population.

In young people at ultra-high-risk, an uncontrolled feasibility case series (SleepWell) with 12 participants (14–24 years) found large effect size improvements in sleep (ISI d = 6.8) following an 8-session individual intervention (Bradley et al., 2018). The treatment included adaptations to account for the developmental age of participants (for example, including parents, incorporating technology) and the associated circadian phase shifts that occur during adolescence (for example, adjusting expectations of sleep timing). Compared to baseline, the confidence intervals indicated improvements in paranoia (GPTS d = 0.6), hallucinatory experiences (SPEQ-H d = 0.3), and negative affect (DASS-21 depression d = 0.5) post-treatment, yet, as appropriate for pilot studies, the *p*-values were not reported (for further statistical rationale see Lancaster et al., 2004). However, the quality of the evidence is limited by the small sample size and lack of control group.

Three studies have evaluated CBTi interventions with patients with persistent psychotic symptoms (two in outpatient settings and one in a residential setting). The Better Sleep Trial (BeST; Freeman et al., 2015) was an assessor-blind pilot randomised controlled trial with 50 patients with current psychotic symptoms. The manualised intervention was delivered on an individual basis over eight sessions. Post-treatment there were large effect size improvements in sleep: 41% of patients in the intervention group no longer had clinical insomnia (compared to 4% in the control group). However, the treatment effect estimation for delusions (PSYRATS d = 0.1 CI −2.0 to 2.6) and hallucinations (PSYRATS d = 0.2, CI −65 to 2.7) included reducing or increasing psychotic experiences. The study was underpowered to determine with any precision the effect on psychotic experiences (which led to the much larger OASIS trial). In addition to the clinical benefits, a health economic evaluation indicated that the sleep intervention may also be cost-effective (Tsiachristas et al., 2018).

In an open label trial of adapted CBTi added to usual care, compared to usual care alone, differential treatment outcomes were observed between different sleep presentations (Chiu et al., 2018). Latent class analysis was conducted on the 74 participants to identify three sleep subtypes: classic severe insomnia (44.6%), insomnia with normal sleep duration (37.8%), insomnia with hypersomnia (17.6%). The greatest treatment benefits were observed in the first cluster. In the hypersomnia cluster, total sleep time reduced and sleep onset latency was halved. A blunted treatment response was reported in the normal sleep duration cluster. However, there were comparable improvements in daytime functioning and overall psychopathology.

Within a residential rehabilitation setting, a four-session CBTi group intervention was evaluated in addition to usual care compared to usual care alone (Hwang et al., 2019)*.* This was a non-randomised, assessor blind trial with 63 patients. No significant changes in psychotic symptoms, depression or anxiety were found, although the study was underpowered. Significant improvements in sleep were reported post-treatment (4 weeks) and at follow-up (8 weeks). However, the effect sizes (ISI d = 0.8 at 8 weeks) were smaller than those reported in the BeST trial (ISI d = 1.9 at 12 weeks). This may indicate that longer treatment duration or individual rather than a group format is important for achieving large treatment effects.

In acute settings, an assessor-blind pilot RCT of a novel sleep intervention has been conducted (Sheaves et al., 2018a). Eighteen (45%) of the 40 participants, recruited from a single ward, had a diagnosis of non-affective psychosis. The intervention included CBTi, sleep monitoring, and light/dark exposure for circadian entrainment. In this acute setting the entire intervention was delivered in 2 weeks. Compared to usual care, there were large effect size reductions in insomnia (ISI d = 0.9) at post-treatment and follow-up (12 weeks). This was the only intervention study to report the outcome on negative symptoms: there was an indication of a small effect size improvement (d = 0.3), however the confidence intervals were wide, including zero, and so the findings are not conclusive. Patients who received the sleep intervention were discharged on average 8.5 days earlier.

One pilot RCT has evaluated imagery rehearsal training (IRT) to reduce nightmare severity in 24 patients with persecutory delusions (Sheaves et al., 2019). In addition to IRT, the intervention included CBT techniques to target identified causal factors for nightmares, for example reducing worry and oversleeping. Large effect size improvements in nightmare severity (DDNSI d = 1.1) and insomnia (SCI d = 1.4) were reported post-treatment (4-weeks) and maintained at follow-up (8-weeks). There was a medium effect size treatment benefit on paranoia favouring the CBT group at both 4 and 8 weeks. However, the confidence intervals are wide, hence a larger trial is required to estimate the effect with greater precision. There was no effect of the treatment on hallucinations which fits with the understanding that psychotic experiences are distinct phenomena with different causal factors (Peralta and Cuesta, 1999; Zavos et al., 2014).

## 4 Discussion

There has been a sharp rise in the number of studies investigating sleep disruption and psychosis: the number of quantitative investigations has doubled in the last five years. There are numerous correlational studies, both in clinical and correlational studies, both in clinical and non-clinical studies, often substantial, that robustly support the co-occurrence of subjective sleep disruption and certain psychotic experiences across the spectrum of psychosis severity. Yet the mechanisms underpinning this relationship remain uncertain. Patient accounts consistently highlight the importance and negative impact of sleep problems and a desire for treatment. The principal advance has been the evaluation of adapted CBT for sleep disorders in patients with non-affective psychosis. The trials have generally been small, limiting the ability to determine the effect of improving sleep on psychotic experiences. Yet taken together an initial clinical picture forms: sleep disturbance is a common and treatable problem across the spectrum of psychosis severity that leads to small improvements in psychotic symptoms such as paranoia and hallucinations. To date, the OASIS trial (Freeman et al., 2017) provides the strongest test, showing that improving insomnia leads to improvements in paranoia and hallucinations. However, studies have been inadequately powered to test this in clinical populations.

There may be differences between how individual psychotic experiences are linked to distinct sleep disorders. In a study of 5000 adolescent twin pairs, the genetic and environmental influences of sleep disturbance were found to overlap with those for paranoia, hallucinations, and cognitive disorganisation but not grandiosity and anhedonia (Taylor et al., 2015). In a robust experimental test of sleep restriction, negative affect was found to mediate the relationship between sleep loss and paranoia but not hallucinations (Reeve et al., 2018a). There are many plausible mechanisms, both psychological and biological, one might expect to underpin the association between sleep dysfunction and psychotic experiences. The most consistent evidence, at a psychological level, is for the role of negative affect mediating the association between insomnia and psychotic experiences (principally paranoia) (Freeman et al., 2009, Freeman et al., 2017; Kasanova et al., 2019; Reeve et al., 2015; Reeve et al., 2018a). Other potential routes include alexithymia, and dissociation, which could be further linked to adversity or trauma. Further work is needed to understand the mechanisms linking hallucinations and insomnia. Studies investigating the interaction of sleep problems with grandiosity, cognitive distortion, or negative symptoms are rare*.* This review has focused on psychological models, as this is the framework underpinning the first-line recommended treatment (CBTi), yet neurodevelopmental and neurochemical mechanisms are also plausible (Lunsford-Avery and Mittal, 2013). For example, abnormalities in the interacting neurotransmitter systems and neural circuits observed in sleep and circadian disruption are also implicated in the aetiology of psychosis (Wulff et al., 2010). At the neurobiological level, overactivity of dopamine receptors are implicated in both the causation of positive psychotic symptoms and wakefulness (Monti and Monti, 2005). To date, we do not have clear evidence to indicate the primary route.

Understanding the mechanistic links will help in the adaptation and improvement of treatment, however it could be argued the key line of research needed now is how to provide the best evidence-based treatments for patients. Psychological interventions for sleep disturbance are applicable, feasible, and demonstrate large effect size improvements in insomnia and nightmares in patients with psychosis. This is consistent with the evidence-base for these treatments in other mental health disorders. The treatment is popular with patients with psychosis: the uptake rate is strikingly high (80–100%) and qualitative data indicate that patients value the opportunity to address sleep disturbance. The studies reporting the largest effect sizes made adaptations to standard treatment protocols. One key adaptation is the use of sleep restriction *principles*, without applying the formal sleep restriction protocol. In practice, this involves a degree of flexibility, sharing the aims of the procedure to reduce time in bed not sleeping and careful negotiation to set a ‘sleep window’. This is particularly important given the experimental evidence for the impact of sleep restriction in the occurrence of psychotic experiences (Reeve et al., 2018a). Screening and evaluation of treatment progress can be achieved using brief self-report measures (for example, Insomnia Severity Index (Morin et al., 2011)) yet they are infrequently used in current practice (Rehman et al., 2017). Treatment protocols outlining adaptions for patients with psychosis have been produced (for example, Sheaves et al., 2018b; Waite et al., 2016b; Waite and Sheaves, 2020; Waters et al., 2017). The next challenge is implementation.

## Role of the funding source

The funder did not have any role in the conduct or reporting of the work.

## Contributors

Felicity Waite drafted the paper. All authors contributed to and have approved the final manuscript.

## Declaration of competing interest

No authors report any conflicts of interest.
